# Impulse oscillometry values calibrated against spirometric obstruction in children with suspected asthma

**DOI:** 10.3389/fped.2026.1746282

**Published:** 2026-06-08

**Authors:** Liang-Mei Lin, Yi-Giien Tsai, Yu-Jun Chang, Jeffrey Eli Whang, Ming-Sheng Lee

**Affiliations:** 1 Pediatric Respiratory Therapy Unit, Changhua Christian Children's Hospital, Changhua, Taiwan; 2 Department of Pediatric Immunology, Changhua Christian Children's Hospital, Changhua, Taiwan; 3 School of Medicine, Kaohsiung Medical University, Kaohsiung, Taiwan; 4 School of Medicine, Chung Shan Medical University, Taichung, Taiwan; 5 Big Data Center, Changhua Christian Hospital, Changhua, Taiwan; 6 Department of Pediatric Pulmonology and Critical Care, Changhua Christian Children's Hospital, Changhua, Taiwan

**Keywords:** bronchodilator test, impulse oscillometry, obstructive lung disease, pediatrics, spirometry

## Abstract

**Background:**

The impulse oscillation system (IOS) requires less cooperation than spirometry for assessing obstructive lung disease in children aged 6–18 years; however, diagnostic criteria for several IOS parameters remain controversial. This study compared IOS and spirometry to determine IOS parameter cutoff values for spirometry-detectable airway obstruction in children.

**Methods:**

Spirometry was used as the reference standard. We enrolled 722 children with suspected asthma. Airway obstruction was defined as a ≥ 12% increase in FEV₁ after bronchodilator or any abnormal spirometric value (FEV₁ < 80% predicted, FEV₁/FVC <80%, or FEF parameters (MMEF, FEF₂₅–₇₅, etc.) < 56% predicted. IOS parameters (R5, X5, AX, Fres and R5–R20) were recorded.

**Results:**

Spirometry and IOS parameters were significantly correlated, R5−R20 correlated the strongest with FEV1 and MMEF. The suggestive AX and Fres cutoff values were 1.54 kPa·L⁻^1^ and 20.41 Hz for 6–11-year olds and 0.54 kPa·L⁻^1^ and 13.77 Hz for 12–18-year olds. After bronchodilator inhalation, Fres and AX decreased respectively by 20% and 40% in 6–11-year olds, predicting obstructive lung diseases with accuracies of 62.2% and 60.2%. In the 12–18-year group, a 20% decrease in Fres and a 40% decrease in AX predicted obstructive lung disease with accuracies of 73.2% and 75.7%, respectively.

**Conclusion:**

Our study compared pre- and post-bronchodilator IOS and spirometry results and identified which IOS values correspond to spirometric obstruction.

## Introduction

Longitudinal lung function assessment is essential for managing pediatric obstructive airway diseases such as asthma. The two main tests are spirometry and impulse oscillometry (IOS). Spirometry, the traditional gold standard for children aged ≥6 years, requires maximal effort and cooperation. In contrast, IOS requires less effort, supporting its expanding clinical use (1, 2).

Predicted lung function values are primarily based on height, with additional influences from age, sex, and race (3). Race- or ethnicity-specific spirometry equations have not been shown to outperform race-neutral equations in predicting pediatric obstructive lung disease (4). Although IOS reference values derived from Caucasian children may be applicable across populations, spirometers and IOS devices used in Asia largely rely on prediction equations based on children of European descent (5, 6). While reference data from healthy Asian children have been reported (7–9), these equations are not yet widely adopted or commercialized. Clinicians also face challenges manually converting IOS measurements into percentage predicted values accounting for race/ethnicity. In this context, using available software may be more practical. However, some IOS parameters, such as resonant frequency (Fres), R5–R20, and reactance area (AX), still lack cutoff values in these programs. This study used pre- and post-bronchodilator spirometry to define airway obstruction and evaluated IOS parameters and their bronchodilator response. Our goal was to identify IOS measures corresponding to spirometry-detectable obstruction, guiding future IOS guidelines and helping pediatricians assess lower airway obstruction in asthmatic Asian children.

## Materials and methods

### Study patients

This was a prospective study that took place from July 2017 to May 2018 at Changhua Christian Hospital in Changhua, Taiwan. We used purposive sampling as our sampling method. Patients aged 6–18 years with suspected or diagnosed asthma who could undergo pre- and post-bronchodilator spirometry were included. All patients were managed by three pediatric physicians trained in asthma care using standardized protocols, in accordance with the GINA 2017 guidelines. Some patients continued controller therapy (such as leukotriene antagonists or inhaled corticosteroids), while others had discontinued treatment due to stable disease. Patients with active pulmonary or upper respiratory infections, acute asthma exacerbations, clinically apparent chest wall deformities, neuromuscular diseases, inability to perform spirometry, or other known respiratory parenchymal diseases were excluded (10). To minimize the impact of medications on pulmonary function, we excluded patients who had used any anticholinergic, theophylline, or beta-agonist within one day prior to testing, or had taken oral or injectable steroids within three days. Adherence to these restrictions was confirmed before testing. However, in order to maintain asthma control and protect patient safety, ongoing inhaled corticosteroids and leukotriene receptor antagonists were continued. We acknowledge that this may introduce a covariate and have noted this limitation accordingly. Because airway size and normal IOS cutoffs vary with age, we followed previous studies and divided participants into two age groups: 6–11 and 12–18 years (11–13).

### Spirometry

Spirometry tests were conducted using a MasterScreen Body/Diffusion pulmonary function device from Jaeger (14). Spirometry followed American Thoracic Society criteria. Children under 10 were excluded if exhalation was <3 s, and those ≥10 if <6 s. At least three reproducible results were required, with the most accurate used for comparison to reference values. Testing sessions were limited to 15 min (15). Common spirometry parameters include forced vital capacity (FVC), forced expiratory volume in the first second (FEV_1_), FEV_1_/FVC ratio, peak expiratory flow rate (PEFR), maximal mid-expiratory flow rate (MMEF), forced expiratory flow at 25% of forced vital capacity (FEF_25_), forced expiratory flow at 50% of forced vital capacity (FEF_50_), forced expiratory flow at 75% of forced vital capacity (FEF_75_), and the ratio between forced expiratory flow between 25% and 75% of vital capacity (FEF_25–75_). Among these, MMEF and FEF25 have proved to be especially valuable for identifying small-airway obstruction (16).

Abnormal spirometry was defined as PEFR or FEV₁ < 80% predicted, or an FEV₁/FVC ratio <80% of the reference value. Additionally, values < 56% of predicted for MMEF, FEF₂₅, FEF₅₀, FEF₇₅, or FEF₂₅–₇₅ (collectively referred to as FEF parameters) were also considered abnormal (10, 17). A positive bronchodilator response was defined as an increase of ≥12% in FEV₁ or ≥40% in any of the FEF parameters. Patients with any of the aforementioned abnormalities in pre- or post-bronchodilator spirometry were classified as having current airway obstruction (16).

### Impulse oscillometry

IOS measurements were performed using a Jaeger MasterScreen Impulse Oscillometry System (Jaeger Co., Würzburg, Germany) during stable tidal breathing with a nose clip and cheek support. Sound impulses were delivered every 0.2 s for 30 s, and respiratory resistance and reactance were measured across frequencies from 5 to 35 Hz. The resistance was determined using pressure and flow signals, with pressure in phase with flow, while the reactance involved pressure out of phase with flow for computation. The relationship between pressure and flow signals allows measurement of the impedance of the total respiratory system (Z5). From this measurement, the total airway resistance (R5), central airway resistance (R20), and reactance (X5) can be calculated. The number in parentheses denotes the measurement frequency. X5 reflects the pressure required to overcome lung viscoelasticity and is considered indicative of peripheral airway function. The R5−R20 (kPa·L⁻^1^·s) could potentially serve as a surrogate for assessing the resistance of small airways. Fres, (Hz) recorded in addition to resistance and reactance, is the frequency at which reactance becomes zero. AX (kPa·L⁻^1^) is an integrated response index for reactance developed by Goldman (18), reflecting the integral of negative reactance values from 5 Hz to Fres. Both Fres and AX may also partly reflect the function of the small airways (3). The reference values used in this study are integrated within the machine. Predicted values for individuals aged 6–11 years were based on the Dencker/Malmberg equations, while those for individuals aged 12–18 years old were based on the Berdel/Lechtenbörger equations (6, 12, 19). During data acquisition, pressure and flow tracings were displayed in real time, and measurements were considered valid only with uninterrupted breathing. Tracings affected by coughing, breath-holding, vocalization, or swallowing were excluded. Quality was also evaluated by monitoring coherence at 5 Hz and 20 Hz, which ideally should be ≥0.7 and ≥0.9, respectively (20). The values for IOS parameters were averaged from three acceptable data collections. Two inhalations of salbutamol (100 µg) were administered via a spacer, and bronchodilator responses were assessed by IOS and spirometry 20 min later. IOS was always conducted prior to spirometry to prevent any influence from the forced maneuver (21).

### Statistical analysis

Data entry and statistical analyses were performed using IBM SPSS Statistics, Version 22.0 (IBM Corp., Armonk, NY, USA). Variables were coded according to study objectives and data types, including demographic and clinical information such as age, sex, height, weight, spirometry, and IOS parameters. The normality of continuous variables was assessed using the Shapiro–Wilk test and Q–Q plots. As most IOS parameters were non-normally distributed, nonparametric methods were applied. Between-group comparisons (obstructive vs. non-obstructive) of basic characteristics were conducted using the Mann–Whitney U test, and results are presented as mean ± SD and interquartile range (IQR), with test statistics and *p*-values explicitly reported in the tables. Correlations between IOS and spirometry indices were assessed using Spearman's rank correlation coefficient (*ρ*), and 95% confidence intervals (CIs) were estimated via Fisher's z transformation. Sensitivity, specificity, positive predictive value (PPV), and negative predictive value (NPV) were calculated, with 95% CIs derived using the Wilson method. To control for multiple testing, *p*-values were adjusted using the Benjamini–Hochberg false discovery rate (FDR) procedure. Receiver operating characteristic (ROC) analyses were performed to evaluate the diagnostic performance of AX, Fres, and R5–R20, with areas under the curve (AUCs) and corresponding 95% CIs reported. Optimal cutoff points were determined using Youden's index. A multivariable logistic regression model combining AX, Fres, and R5–R20 was constructed and evaluated. To assess model robustness and generalizability, we conducted internal validation using bootstrap optimism correction (1,000 resamples) and temporal validation based on enrollment order. The sample size was calculated using G*Power with a z-test set at a statistical power of 0.8, *α* level of 0.05, and effect size of 0.3, yielding an estimated sample size of 178 participants. The study was approved by the Institutional Review Board of Changhua Christian Children's Hospital (IRB No. 170320). Informed consent was obtained from the participants before data collection.

## Results

A total of 722 children suspected of obstructive lung disease were included: 569 aged 6–11 years (266 females, 303 males) and 153 aged 12–18 years (65 females, 88 males). Obstructive spirometry was observed in 184 children (32.3%) and 36 adolescents (23.5%), respectively. Patient characteristics are summarized in Table 1. The body mass index of patients aged 6–11 years with obstructive spirometry results was significantly higher, 19.06 ± 4.52 compared to 18.2 ± 3.67 for patients without obstructive spirometry results.

**Table 1:** General characteristics of the patients included in this study classified by spirometry results.

Characteristics	Age: 6–11 (*N* = 569)					Age: 12–18 (*N* = 153)				
	Normal (*N* = 385)		Obstructive (*N* = 184)		*p*-value	Normal (*N* = 117)		Obstructive (*N* = 36)		*p*-value
	Mean ± SD	Median (IQR)	Mean ± SD	Median (IQR)		Mean ± SD	Median (IQR)	Mean ± SD	Median (IQR)	
Age	8.83 ± 1.67	9 (7–10)	8.82 ± 1.58	9 (8–10)	0.884	12.61 ± 1.08	12 (12–13)	12.83 ± 1.34	12 (12–13)	0.359
Height	135.42 ± 12.10	135.9 (126–144)	135.67 ± 11.75	136 (127.05–144)	0.795	157.19 ± 6.78	157 (154–161.5)	156.99 ± 9.03	157.5 (150.6–162.9)	0.705
Body weight	34.26 ± 11.79	31 (26–40)	36.04 ± 13.40	33.05 (26.55–41.35)	0.135	51.23 ± 11.78	49.9 (43–56)	49.93 ± 11.25	48.55 (42.45–54.5)	0.577
BMI	18.20 ± 3.67	17.10 (15.38–20.20)	19.06 ± 4.52	18.00 (16.05–21.01)	0.019	20.59 ± 3.79	19.60 (17.80–22.10)	20.14 ± 3.42	19.66 (17.81–22.13)	0.624
FVC <80%, N (%)	22 (5.7%)		31 (16.84%)		<0.001	11 (9.40%)		14 (38.89%)		<0.001

BMI, body mass index; SD, standard deviation; IQR, interquartile range represents the distance between the 25th percentile and 75th percentile; FVC, forced vital capacity.

*p*-values were calculated using the Mann–Whitney U Test.

### Correlations between IOS and spirometry results

Correlations between spirometry and IOS parameters for children aged 6–11 and 12–18 years are presented separately in Tables 2, 3, respectively. Correlations between spirometry and IOS parameters were significant but generally weak to moderate. In both age strata, R5, R10, X5, R5−R20, Fres, and AX were all significantly correlated with spirometric indices, including PEF, FEV₁, FEV₁/FVC, MMEF, and MEF₂₅–₇₅. Among children aged 6–11 years, FEV₁ was best correlated with X5 (r = −0.333), FEV₁/FVC was best correlated with R5 and R5–R20 (r = −0.319), and the strongest overall correlation was observed between MMEF and R5–R20 (r = −0.408). In the 12–18-year age group, FEV₁ was best correlated with X5 (r = −0.310), FEV₁/FVC was best correlated with Fres (r = −0.453), and the strongest correlation was observed between MEF₂₅ and Fres (r = −0.491). When considering FEF₂₅ as the optimal spirometric marker for detecting small-airway disease, the top three IOS parameters most strongly correlated with FEF₂₅ in children aged 6–11 years were R5−R20, AX, and R5. For children aged 12–18 years, the top three were Fres, R5−R20, and AX. Correlations between post-bronchodilator changes in IOS parameters and spirometry indices are presented in Supplementary Tables S1 and 2. In children aged 6–11 years, the strongest correlation was observed between changes in AX and FEV₁/FVC, whereas in the 12–18-year age group, changes in AX showed the strongest correlation with MMEF.

**Table 2:** The correlations between IOS parameters and spirometry results for children aged 6 to 11 years.

Age: 6–11	(*n* = 569)	FVC	PEF	FEV1	FEV1/FVC	MMEF	MEF25	MEF50	MEF75	Change%-FEV1
R5 (% predicted)	*ρ*	−0.087*	−0.235†	−0.272†	−0.319†	−0.390†	−0.373†	−0.374†	−0.304†	0.135*
	(95% CI)	(−0.168, −0.005)	(−0.311, −0.156)	(−0.346, −0.194)	(−0.391, −0.243)	(−0.458, −0.318)	(−0.442, −0.300)	(−0.443, −0.301)	(−0.377, −0.227)	(0.033, 0.235)
R10 (% predicted)	*ρ*	−0.047	−0.190†	−0.218†	−0.298†	−0.346†	−0.332†	−0.334†	−0.262†	0.114*
	(95% CI)	(−0.129, 0.035)	(−0.268, −0.110)	(−0.295, −0.138)	(−0.371, −0.221)	(−0.416, −0.272)	(−0.403, −0.257)	(−0.405, −0.259)	(−0.337, −0.184)	(0.011, 0.214)
X5 (% predicted)	*ρ*	−0.213†	−0.165†	−0.333†	−0.133**	−0.303†	−0.275†	−0.294†	−0.244†	0.154**
	(95% CI)	(−0.290, −0.133)	(−0.244, −0.084)	(−0.404, −0.258)	(−0.213, −0.051)	(−0.376, −0.226)	(−0.349, −0.197)	(−0.367, −0.217)	(−0.320, −0.165)	(0.052, 0.253)
R5−R20 (kPa·L⁻^1^·s)	*ρ*	−0.126**	−0.278†	−0.315†	−0.319†	−0.408†	−0.390†	−0.388†	−0.332†	0.217†
	(95% CI)	(−0.206, −0.044)	(−0.352, −0.200)	(−0.387, −0.239)	(−0.391, −0.243)	(−0.474, −0.337)	(−0.458, −0.318)	(−0.456, −0.316)	(−0.403, −0.257)	(0.117, 0.313)
Fres (Hz)	*ρ*	−0.022	−0.183†	−0.207†	−0.282†	−0.340†	−0.350†	−0.328†	−0.250†	0.240†
	(95% CI)	(−0.104, 0.060)	(−0.261, −0.102)	(−0.284, −0.127)	(−0.356, −0.205)	(−0.411, −0.265)	(−0.42, −0.276)	(−0.399, −0.253)	(−0.326, −0.171)	(0.141, 0.335)
AX (kPa·L⁻^1^)	*ρ*	−0.023	−0.224†	−0.218†	−0.306†	−0.375†	−0.386†	−0.363†	−0.299†	0.281†
	(95% CI)	(−0.105, 0.059)	(−0.301, −0.144)	(−0.295, −0.138)	(−0.379, −0.230)	(−0.444, −0.302)	(−0.454, −0.314)	(−0.432, −0.289)	(−0.372, −0.222)	(0.184, 0.373)

*ρ* = Spearman's rank correlation coefficient; *, **, and ^†^represent significance levels of *p* < 0.05, *p* < 0.01, and *p* < 0.001, respectively.

**Table 3:** The correlations between IOS parameters and spirometry results for children aged 12 to 18 years.

Age: 12–18	(*n* = 153)	FVC	PEF	FEV1	FEV1/FVC	MMEF	MEF25	MEF50	MEF75	Change%−FEV1
R5 (% predicted)	*ρ*	−0.081	−0.338†	−0.292†	−0.336†	−0.434†	−0.424†	−0.393†	−0.412†	0.256*
	(95% CI)	(−0.236, 0.079)	(−0.471, −0.189)	(−0.431, −0.14)	(−0.470, −0.187)	(−0.554, −0.296)	(−0.546, −0.284)	(−0.519, −0.25)	(−0.536, −0.271)	(0.049, 0.442)
R10 (% predicted)	*ρ*	−0.084	−0.340†	−0.275†	−0.311†	−0.392†	−0.369†	−0.371†	−0.416†	0.209
	(95% CI)	(−0.240, 0.075)	(−0.473, −0.192)	(−0.416, −0.122)	(−0.448, −0.160)	(−0.518, −0.249)	(−0.498, −0.223)	(−0.500, −0.226)	(−0.539, −0.276)	(0.000, 0.401)
X5 (% predicted)	*ρ*	−0.211**	−0.312†	−0.310†	−0.166*	−0.252**	−0.237**	−0.223**	−0.316†	0.185
	(95% CI)	(−0.358, −0.054)	(−0.448, −0.161)	(−0.447, −0.159)	(−0.316, −0.008)	(−0.395, −0.097)	(−0.381, −0.081)	(−0.369, −0.067)	(−0.452, −0.166)	(−0.025, 0.380)
R5− R20 (kPa·L⁻^1^·s)	*ρ*	−0.071	−0.237**	−0.289†	−0.366†	−0.442†	−0.484†	−0.396†	−0.311†	0.299**
	(95% CI)	(−0.227, 0.088)	(−0.381, −0.081)	(−0.428, −0.137)	(−0.496, −0.220)	(−0.561, −0.305)	(−0.597, −0.352)	(−0.522, −0.253)	(−0.448, −0.160)	(0.096, 0.478)
Fres (Hz)	*ρ*	−0.049	−0.206*	−0.277†	−0.453†	−0.433†	−0.491†	−0.381†	−0.304†	0.178
	(95% CI)	(−0.206, 0.110)	(−0.353, −0.049)	(−0.417, −0.124)	(−0.571, −0.317)	(−0.554, −0.295)	(−0.603, −0.360)	(−0.509, −0.237)	(−0.441, −0.153)	(−0.032, 0.374)
AX (kPa·L⁻^1^)	*ρ*	−0.060	−0.205*	−0.276†	−0.429†	−0.412†	−0.48†	−0.34†	−0.287†	0.202
	(95% CI)	(−0.217, 0.099)	(−0.352, −0.048)	(−0.416, −0.123)	(−0.550, −0.290)	(−0.536, −0.271)	(−0.593, −0.348)	(−0.473, −0.192)	(−0.426, −0.134)	(−0.008, 0.395)

*ρ* = Spearman's rank correlation coefficient; *, **, and ^†^represent significance levels of *p* < 0.05, *p* < 0.01, and *p* < 0.001, respectively.

### Results of R5, X5, and bronchodilator test of IOS parameters in diagnosing airway obstruction

We assessed the accuracy of diagnosing obstructive spirometry using R5 and X5, with reference values provided by the lung function machine. The results for children aged 6–11 years and 12–18 years are presented separately in Tables 4, 5 and Supplementary Table S3. Based on our literature review, we selected two indicators—R5 (% predicted) > 150% and X5 (% predicted) > 150%—to predict the presence of obstructive lung disease in children (3, 22). However, not many patients met these two criteria. Of the 569 patients aged 6–11 years, only three met the R5 (% predicted) > 150% criterion and only five met the X5 > 150% criterion. Similarly, of the 153 patients aged 12–18 years, only five met either the R5 > 150% or X5 > 150% criterion. Due to the limited sample size, although the specificity of these two indicators is high, only X5 > 150% for the 6–11-year age group reached statistical significance. Additionally, to facilitate interpretation and clinical application of bronchodilator test results across different parameters, we evaluated changes within a 10%–40% range, based on previously published studies (3, 5, 23, 24). As shown in Tables 4, 5, we propose several suitable cutoff points for the bronchodilator test: a ≥ 20% reduction in R5, X5, or Fres, and a ≥ 40% reduction in AX or R5–R20 following bronchodilator inhalation to identify obstructive spirometry results. All parameters reached statistical significance. Of these, a decrease in R5 > 20% showed the highest specificity, with values of 0.779 for patients aged 6–11 years and 0.872 for patients aged 12–18 years. Supplementary Tables S4 and 5 also provide results for a 10% and 40% reduction in R5 and X5, a 10% and 20% reduction in R5−R20, and a 30% reduction in AX after bronchodilator inhalation for diagnosing obstructive spirometry results. To provide a more complete presentation of our data, the spirometry-defined small- and large-airway obstruction subgroups and bronchodilator responsiveness within these obstructive subgroups are shown in Supplementary Tables S6 and 7.

**Table 4:** The accuracy of diagnosing obstructive spirometry results using IOS parameters for children aged 6 to 11 years.

Age: 6–11		Normal** N (%)	Obstructive** N (%)	Total N (%)	*p*-value (FDR)	Sensitivity (95% CI)	Specificity (95% CI)	PPV (95% CI)	NPV (95% CI)
R5 (% predicted)> 150%	No	384 (99.7)	182 (98.9)	566 (99.5)	0.246	0.011	0.997	0.667	0.678
	Yes	1 (0.3)	2 (1.1)	3 (0.5)		(0.003–0.039)	(0.985–1.000)	(0.208–0.939)	(0.639–0.716)
X5 (% predicted)> 150%	No	384 (99.7)	180 (97.8)	564 (99.1)	0.046	0.022	0.997	0.800	0.681
	Yes	1 (0.3)	4 (2.2)	5 (0.9)		(0.008–0.055)	(0.985–1.000)	(0.376–0.964)	(0.641–0.718)
R5 decrease > 20%*	No	300 (77.9)	121 (65.8)	421 (74.0)	0.005	0.342	0.779	0.426	0.713
	Yes	85 (22.1)	63 (34.2)	148 (26.0)		(0.278–0.414)	(0.735–0.818)	(0.349–0.506)	(0.668–0.754)
X5 decrease > 20%*	No	269 (69.9)	106 (57.6)	375 (65.9)	0.007	0.424	0.699	0.402	0.717
	Yes	116 (30.1)	78 (42.4)	194 (34.1)		(0.355–0.496)	(0.651–0.742)	(0.336–0.472)	(0.670–0.761)
R5−R20 decrease > 40%*	No	278 (72.2)	115 (62.5)	393 (69.1)	0.027	0.375	0.722	0.392	0.707
	Yes	107 (27.8)	69 (37.5)	176 (30.9)		(0.308–0.447)	(0.675–0.764)	(0.323–0.466)	(0.661–0.750)
Fres decrease > 20%*	No	281 (73.0)	111 (60.3)	392 (68.9)	0.005	0.397	0.730	0.412	0.717
	Yes	104 (27.0)	73 (39.7)	177 (31.1)		(0.329–0.469)	(0.683–0.772)	(0.343–0.486)	(0.670–0.759)
AX decrease > 40%*	No	246 (64.1)	88 (47.8)	334 (58.8)	0.002	0.522	0.641	0.410	0.737
	Yes	138 (35.9)	96 (52.2)	234 (41.2)		(0.450–0.593)	(0.591–0.687)	(0.349–0.474)	(0.687–0.781)

*indicates a decrease in absolute difference before and after inhalation of bronchodilators, regardless of sign; **represents the results of spirometry tests; *p*-values were calculated using the Chi-square test or Fisher's exact test, depending on cell counts, and were adjusted for multiple comparisons using the Benjamini-Hochberg false discovery rate (FDR) correction.

**Table 5:** The accuracy of diagnosing obstructive spirometry results using IOS parameters for children aged 12 to 18 years.

Age: 12–18		Normal** N (%)	Obstructive** N (%)	Total N (%)	*p*-value (FDR)	Sensitivity (95% CI)	Specificity (95% CI)	PPV (95% CI)	NPV (95% CI)
R5 (% predicted)> 150%	No	115 (98.3)	33 (91.7)	148 (96.7)	0.085	0.083	0.983	0.600	0.777
	Yes	2 (1.7)	3 (8.3)	5 (3.3)		(0.029–0.218)	(0.940–0.995)	(0.231–0.882)	(0.703–0.837)
X5 (% predicted)> 150%	No	115 (98.3)	33 (91.7)	148 (96.7)	0.085	0.083	0.983	0.600	0.777
	Yes	2 (1.7)	3 (8.3)	5 (3.3)		(0.029–0.218)	(0.940–0.995)	(0.231–0.882)	(0.703–0.837)
R5 decrease > 20%*	No	102 (87.2)	21 (58.3)	123 (80.4)	<0.001	0.417	0.872	0.500	0.829
	Yes	15 (12.8)	15 (41.7)	30 (19.6)		(0.271–0.578)	(0.799–0.921)	(0.332–0.668)	(0.753–0.886)
X5 decrease > 20%*	No	95 (81.2)	23 (63.9)	118 (77.1)	0.043	0.361	0.812	0.371	0.805
	Yes	22 (18.8)	13 (36.1)	35 (22.9)		(0.225–0.524)	(0.732–0.872)	(0.232–0.537)	(0.724–0.866)
R5−R20 decrease > 40%*	No	71 (62.3)	12 (33.3)	83 (55.3)	0.004	0.667	0.623	0.358	0.855
	Yes	43 (37.7)	24 (66.7)	67 (44.7)		(0.503–0.798)	(0.531–0.706)	(0.254–0.478)	(0.764–0.915)
Fres decrease > 20%*	No	95 (81.2)	19 (52.8)	114 (74.5)	0.001	0.472	0.812	0.436	0.833
	Yes	22 (18.8)	17 (47.2)	39 (25.5)		(0.320–0.630)	(0.732–0.872)	(0.293–0.590)	(0.754–0.891)
AX decrease > 40%*	No	95 (81.2)	15 (42.9)	110 (72.4)	<0.001	0.571	0.812	0.476	0.864
	Yes	22 (18.8)	20 (57.1)	42 (27.6)		(0.409–0.720)	(0.732–0.872)	(0.334–0.623)	(0.787–0.916)

*indicates a decrease in absolute difference before and after inhalation of bronchodilators, regardless of sign; **represents the results of spirometry tests; *p*-values were calculated using the Chi-square test or Fisher's exact test, depending on cell counts, and were adjusted for multiple comparisons using the Benjamini-Hochberg false discovery rate (FDR) correction.

### Results of AX, fres, and R5−R20 at various diagnostic cutoff points

**Figure 1:**
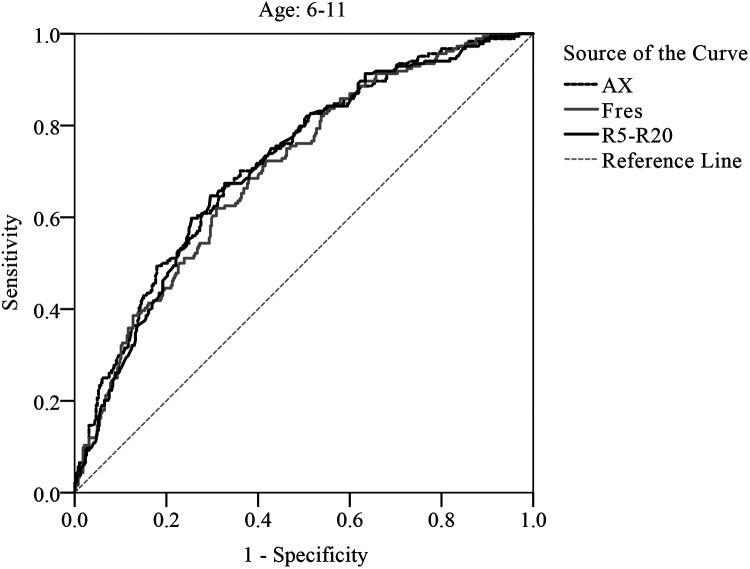
ROC curves for AX, fres, and R5−R20 for diagnosing obstructive lung disease in children aged 6–11 years.

**Figure 2:**
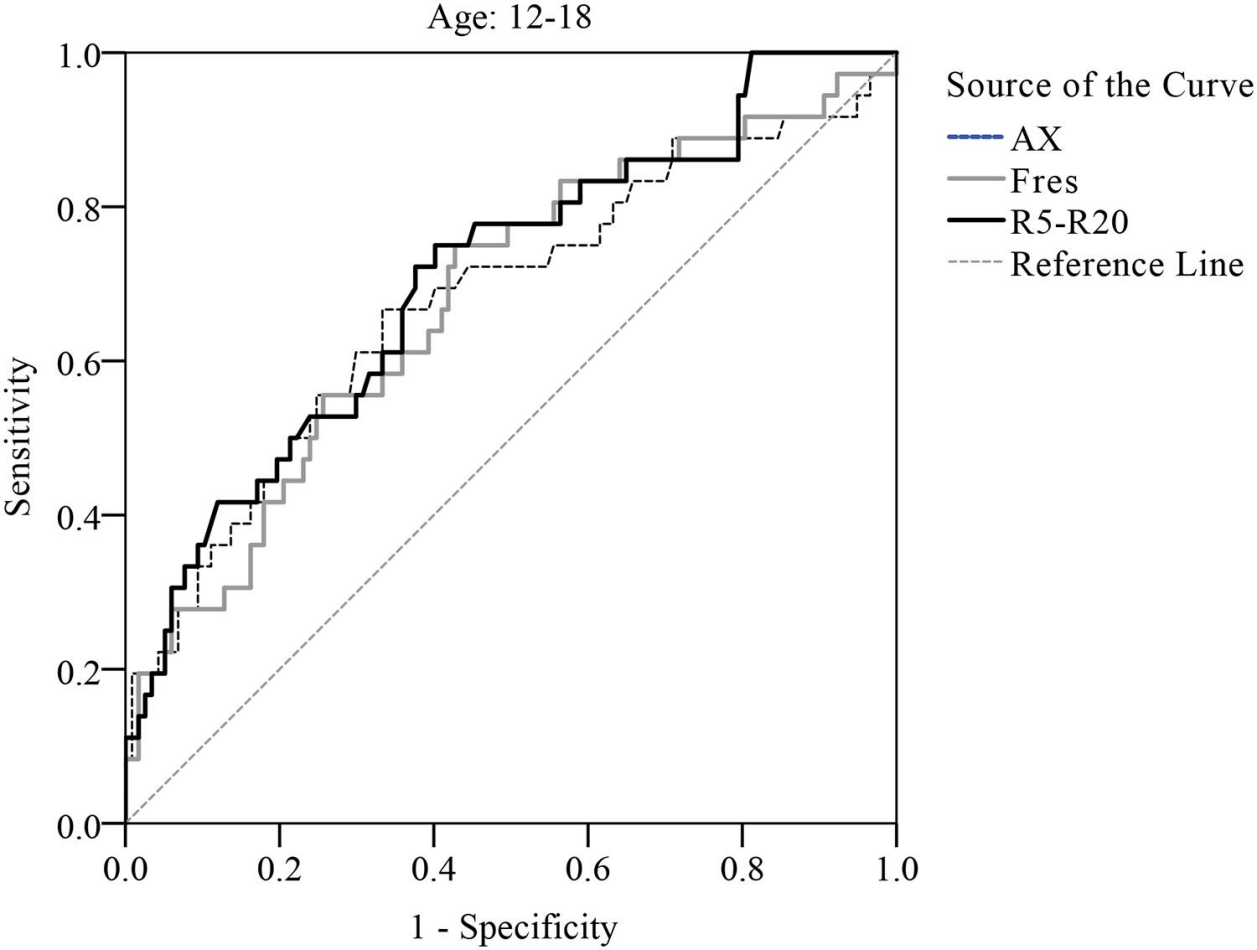
ROC curves for AX, fres, and R5−R20 for diagnosing obstructive lung disease in children aged 12–18 years.

Most previous studies have not clearly specified the normal cutoff values for AX, Fres, and R5−R20 for children aged 6–11 years or 12–18 years. By using Youden's index, the optimal cutoff values for AX, Fres, and R5−R20 were 1.535 kPa·L⁻^1^, 20.405 Hz, and 0.185 kPa·L⁻^1^·s, respectively, for children aged 6–11 years and 0.538 kPa·L⁻^1^, 13.772 Hz, and 0.062 kPa·L⁻^1^·s, respectively, for those aged 12–18 years. In addition to these cutoff values, we provide cutoff values with specificities close to 85% and 95% in Table 6. We plotted ROC curves for AX, Fres, and R5−R20 for diagnosing obstructive lung disease and provided the AUC for these parameters in Figures 1, 2. For children aged 6–11 years, the ROC AUC for AX, Fres, and R5−R20 was 0.721, 0.704, and 0.714, respectively. For children aged 12–18 years, the ROC AUC for AX, Fres, and R5−R20 was 0.677, 0.678, and 0.707, respectively. A multivariable logistic regression model combining AX, Fres, and R5–R20 was further evaluated in both age strata, with results presented in Supplementary Table S8. Given the higher BMI in the obstructive 6–11-year group, we performed multivariable logistic regression adjusting for age, BMI, height, and sex. Adjusted AUCs were 0.711 for AX, 0.692 for Fres, and 0.707 for R5–R20, with corresponding ORs in Supplementary Table S9. Minimal *Δ*AUC (≈0.01–0.02) and consistent significance indicate BMI and age had little confounding effect on diagnostic performance. Internal (bootstrap) and temporal validation analyses for both age groups (6–11 and 12–18 years) are presented in Supplementary Table S10, demonstrating nearly identical corrected and original AUCs, which confirms excellent internal stability of the diagnostic models. Finally, to further validate the reliability of our data, we defined a small-airway–only (SAO-only) group as patients with spirometric parameters of PEFR, FEV₁, and FEV₁/FVC all ≥ 80% predicted, but with at least one FEF parameter < 56% predicted. We then reported descriptive statistics for AX, Fres, and R5–R20 in the SAO-only group and compared them with those of the non-obstructive group in Supplementary Table S11.

**Table 6:** Diagnostic performance of AX, fres, R5–R20, and multivariable models for predicting abnormal airway resistance among children aged 6–11 years and 12–18 years.

Age	Variable	Cut-off value	AUC (95% CI)	*p*-value	Sensitivity (95% CI)	Specificity (95% CI)	PPV (95% CI)	NPV (95% CI)
6–11 years	AX (kPa·L⁻^1^)	≥1.535*	0.721 (0.678–0.765)	<0.001	0.668 (0.598–0.732)	0.673 (0.624–0.718)	0.494 (0.432–0.556)	0.809 (0.763–0.849)
		≥2.197	0.721 (0.678–0.765)	<0.001	0.429 (0.360–0.502)	0.849 (0.810–0.882)	0.577 (0.493–0.656)	0.757 (0.714–0.795)
		≥3.001	0.721 (0.678–0.765)	<0.001	0.201 (0.150–0.265)	0.951 (0.924–0.968)	0.661 (0.530–0.771)	0.713 (0.673–0.751)
	Fres (Hz)	≥20.405*	0.704 (0.659–0.749)	<0.001	0.620 (0.548–0.687)	0.691 (0.643–0.735)	0.489 (0.426–0.553)	0.792 (0.745–0.832)
		≥22.78	0.704 (0.659–0.749)	<0.001	0.397 (0.329–0.469)	0.849 (0.810–0.882)	0.557 (0.472–0.639)	0.747 (0.704–0.785)
		≥25.063	0.704 (0.659–0.749)	<0.001	0.147 (0.103–0.205)	0.948 (0.921–0.966)	0.574 (0.433–0.705)	0.699 (0.659–0.737)
	R5–R20 (kPa·L⁻^1^·s)	≥0.185*	0.714 (0.670–0.758)	<0.001	0.674 (0.603–0.737)	0.673 (0.624–0.718)	0.496 (0.435–0.558)	0.812 (0.765–0.851)
		≥0.250	0.714 (0.670–0.758)	<0.001	0.391 (0.324–0.463)	0.839 (0.799–0.872)	0.537 (0.453–0.620)	0.743 (0.699–0.781)
		≥0.344	0.714 (0.670–0.758)	<0.001	0.136 (0.094–0.193)	0.951 0.924–0.968)	0.568 (0.422–0.703)	0.697 (0.657–0.735)
12–18 years	AX (kPa·L⁻^1^)	≥0.538*	0.677 (0.568–0.787)	0.001	0.667 (0.503–0.798)	0.667 (0.577–0.746)	0.381 (0.271–0.504)	0.867 (0.781–0.922)
		≥0.976	0.677 (0.568–0.787)	0.001	0.389 (0.248–0.551)	0.855 (0.780–0.907)	0.452 (0.292–0.622)	0.820 (0.742–0.878)
		≥1.491	0.677 (0.568–0.787)	0.001	0.194 (0.098–0.350)	0.957 (0.904–0.982)	0.583 (0.320–0.807)	0.794 (0.720–0.853)
	Fres (Hz)	≥13.772*	0.678 (0.573–0.782)	0.001	0.750 (0.589–0.862)	0.573 (0.482–0.659)	0.351 (0.253–0.462)	0.882 (0.790–0.936)
		≥18.997	0.678 (0.573–0.782)	0.001	0.306 (0.180–0.469)	0.855 (0.780–0.907)	0.393 (0.236–0.576)	0.800 (0.721–0.861)
		≥20.941	0.678 (0.573–0.782)	0.001	0.194 (0.098–0.350)	0.949 (0.893–0.976)	0.538 (0.291–0.768)	0.793 (0.718–0.852)
	R5–R20 (kPa·L⁻^1^·s)	≥0.062*	0.707 (0.607–0.806)	<0.001	0.750 (0.589–0.862)	0.598 (0.508–0.683)	0.365 (0.264–0.479)	0.886 (0.797–0.939)
		≥0.138	0.707 (0.607–0.806)	<0.001	0.417 (0.271–0.578)	0.855 (0.780–0.907)	0.469 (0.309–0.636)	0.826 (0.749–0.884)
		≥0.198	0.707 (0.607–0.806)	<0.001	0.194 (0.098–0.350)	0.949 (0.893–0.976)	0.538 (0.291–0.768)	0.793 (0.718–0.852)

*Best cutoff values derived from Youden's index; AUC, area under the curve; PPV, positive predictive value; NPV, negative predictive value; CI, confidence interval.

## Discussion

The primary aim of this study was to determine cutoff values for IOS parameters corresponding to spirometry-defined airway obstruction, which have not been clearly established in Asian populations. Traditionally, establishing lung function reference values requires IOS data from large cohorts of healthy children across age groups to define 95% confidence intervals, often necessitating nearly 1,000 participants per age group (25). To overcome this challenge, we used the well-established spirometry test as the gold standard to further evaluate and develop cutoff values for IOS parameters. This approach has also been previously used to determine the bronchodilator response of IOS in elderly patients with chronic obstructive airway disease (26). Additionally, we adopted more stringent spirometry criteria for diagnosing small-airway obstruction. Abnormal FEF parameters, which are commonly used to assess small-airway obstruction, were defined as values below 56% of the predicted value, whereas some studies have used a threshold of <65% (27). By adopting a stricter cutoff, we aimed to reduce measurement variability, minimize false-positive classifications, and ensure that the derived IOS parameters represent a conservative lower-bound diagnostic threshold. To avoid missing patients with mild small-airway obstruction due to this stricter cutoff, an additional criterion of >40% improvement in small-airway parameters after bronchodilator administration was applied as a complementary measure (16, 28, 29).

Previous studies have suggested that R5 or X5 > 150% of the predicted indicates abnormality and may help diagnose obstructive lung disease (3, 30). However, few participants met this criterion in our study, likely because our cohort consisted mainly of stable outpatients rather than patients with acute exacerbations. Among the 722 participants, only four had an FEV1 < 60% of predicted, suggesting that most outpatient follow-up patients may not exhibit R5 or X5 values >150% of predicted. Instead, alternative small-airway markers such as Fres, AX, or R5−R20 might offer more reliable insights into their condition (27). As shown in Figures 1, 2, the three small-airway markers demonstrated good AUC (approaching 0.7) in predicting obstructive lung disease.

In this study, spirometry and IOS parameters showed statistically significant but weak-to-moderate correlations, with all correlation coefficients below 0.5. These might suggest that while the two tests are correlated, they are not entirely interchangeable. From the perspective of detection principles, IOS uses sound waves to measure airway pressure and flow across frequencies, allowing calculation of characteristics such as resistance. Unlike spirometry, performed with forceful exhalation, IOS is done during quiet breathing and more directly reflects lung-specific changes—elasticity, compliance, and airway obstruction—while spirometry reflects both pulmonary changes and respiratory muscle function (28). In patients with mixed conditions, both IOS and spirometry provide complementary information. For example, in patients with scoliosis or pectus excavatum and concomitant asthma, spirometry may demonstrate combined restrictive and obstructive patterns. In such cases, IOS may better reflect asthma control with less influence from chest wall structural abnormalities, allowing a more accurate assessment of asthma management. IOS and spirometry are like two mirrors placed at different positions on the patient, and the performance of both tests would allow a clearer and more comprehensive view of the patient's asthma control (30, 31). Furthermore, as shown in Tables 2, 3, IOS parameters correlated more strongly with spirometric indices of small-airway function (MMEF, MEF25, MEF50, and MEF75) than with large-airway indices (FEV1 and PEFR), supporting the utility of IOS in assessing distal small-airway obstruction (32).

One of the key contributions of this study is our effort to establish cutoff reference values for bronchodilator response in IOS parameters. According to the European Respiratory Society (ERS) technical standards, a ≥ 40% decrease in R5, a 40%–50% change in X5, and an ≥80% decrease in AX are considered indicative of a positive bronchodilator response in IOS assessments (12, 33, 34). As shown in Supplementary Tables S4 and 5, the number of patients who exhibited a > 40% change in R5 or X5 was relatively small across both age groups, suggesting that such stringent criteria may have limited applicability in routine clinical practice. Instead, a 20% decrease in R5 and X5 may provide an acceptable balance of sensitivity and specificity and could be considered a meaningful marker for both age groups. Our findings are consistent with the suggestion by Song et al., who reported that a 20% decrease in R5 following bronchodilator administration can detect minimal changes in airway caliber, even in healthy children (35). We propose that a 40% decrease in AX after bronchodilator inhalation may define a positive response, yielding diagnostic accuracies of 60.2% and 75.7% for the 6–11 and 12–18-year age groups, respectively. Previous studies have also suggested lower cutoff values for AX; for example, Komarow et al. reported that a 29.1% decrease in AX following bronchodilator administration could effectively identify asthma in school-aged children (36). Additionally, in studies involving older adults, Park et al. (2019) proposed bronchodilator response cutoffs of 18.2% for R5, 18.8% for Fres, 15.4% for R5−R20, and 36.1% for AX. Most of these values closely align with our study results, except for R5−R20, for which we suggest using a 40% cutoff (26). Review of Supplementary Tables S4 and 5 showed that a 10%–20% bronchodilator response in R5–R20 had <50% specificity in our pediatric patients. This may reflect greater airway instability in children and the high sensitivity of R5–R20 for small-airway obstruction (27).

Another key contribution of this study is the establishment of reference ranges for Fres, AX, and R5–R20 in children aged 6–18 years, for which normal values had not been clearly defined in pediatric populations. Adult patients' normal AX is typically <0.33 kPa·L⁻^1^, while the normal R5−R20 is usually < 0.07 kPa·L⁻^1^·s (20, 25). Lin et al. previously collected IOS data from 140 healthy Asian controls and defined cutoff values for Fres, AX, and R5−R20 for children aged 6–11 years as 23.88 Hz, 2.30 kPa·L⁻^1^, and 0.29 kPa·L⁻^1^·s, respectively. When these healthy control-derived reference values were compared with spirometry results for our patients aged 6–11 years (Table 6), the specificity was estimated to range between 85% and 95% (37). Additionally, a team from the University of California analyzed a smaller cohort of patients (*n* = 121) with a mean age of 12 years and reported cutoff values for AX and R5−R20 of 9.5 cm H₂O·L⁻^1^ (0.93 kPa·L⁻^1^) and 1.5 cm H₂O·L⁻^1^·s (0.147 kPa·L⁻^1^·s), respectively (32). These findings also align with our study results for the 12–18 age group (Table 6), with specificity approaching 85%. In summary, our suggested values for Fres, AX, and R5−R20 were 20.405 Hz, 1.535 kPa·L⁻^1^, and 0.185 kPa·L⁻^1^·s for children (6–11 years) and 13.772 Hz, 0.538 kPa·L⁻^1^, and 0.062 kPa·L⁻^1^·s for adolescents (12–18 years), respectively. The proposed cut-off values above should be interpreted primarily as thresholds for identifying spirometry-defined airway obstruction that is predominantly driven by mixed airway involvement rather than isolated small-airway disease. Accordingly, they represent general markers for detecting airflow obstruction rather than phenotype-specific indicators capable of distinguishing proximal from distal airway involvement. These cut-off values are not intended to differentiate large-airway obstruction (LAO) from small-airway obstruction (SAO), nor to determine the degree of airflow limitation. Further refinement of IOS thresholds across distinct obstructive phenotypes will require larger cohorts specifically powered to analyze SAO-only and LAO-only populations separately.

Our study has several limitations. First, only 153 participants were recruited in the 12–18-year age group, slightly below the estimated requirement of 178, which may limit representativeness. This reflects the difficulty of enrolling adolescents, as asthma often stabilizes during this period. Second, patients with acute exacerbations were excluded, so most had mild symptoms, which may limit generalizability. However, severe exacerbations are unsuitable for lung function testing, and our aim was to identify IOS markers of spirometry-detectable obstruction, particularly in mild or subclinical disease, making this exclusion unlikely to affect our conclusions. Third, since spirometry served as the reference standard and IOS is generally regarded as more sensitive for detecting small-airway disease, our study design might have resulted in misclassification. Children with abnormal IOS but normal spirometry findings may have been classified as “non-obstructive,” which could underestimate the true diagnostic capability of IOS. Hence, the proposed cutoff values should be regarded as practical thresholds relative to spirometry rather than true normative IOS reference values (38). Fourth, patients with mild or subtle chest wall structural abnormalities may not have been completely identified and excluded during the recruitment period, which could affect the interpretation of spirometry results. Future studies incorporating lung volume measurements are warranted. Finally, the American Thoracic Society and European Respiratory Society have recently recommended the use of lower limit of normal (LLN) and z-scores instead of % predicted or fixed cutoffs. Although this recommendation has not yet been widely adopted in the management of pediatric asthma, future studies could explore how our IOS-derived suggestive cutoffs perform when spirometric obstruction is defined using LLN based criteria rather than % predicted or fixed thresholds (22, 29, 39). The strength of this study lies in the inclusion of 569 participants in the 6–11-year age group, which helped minimize individual variability. In addition, we performed bootstrap and temporal validations to demonstrate that our diagnostic thresholds were stable and reproducible, confirming the model's internal generalizability. Our findings are consistent with previous pediatric studies and may serve as a practical reference for clinicians using IOS to assess lower airway obstruction in school-aged children.

## Conclusion

This study compared IOS with spirometry to establish cutoff values and identify IOS parameters corresponding to spirometric obstruction in Asian children. While R5 and X5 are commonly used, Fres, AX, and R5–R20 also have diagnostic value. Baseline and bronchodilator-induced changes in these parameters effectively reflect airway obstruction and may aid outpatient monitoring. Given its minimal dependence on respiratory muscle strength, IOS offers more direct airway measurements than spirometry and complements its use in evaluating pediatric asthma. However, because airway growth and lung function change continuously with height, dividing participants into two age groups cannot fully address height-related variability. Thus, the proposed cut-off values should be viewed as pragmatic, spirometry-calibrated thresholds derived from a real-world pediatric asthma cohort rather than definitive normative standards. Future large-scale studies are needed to develop height-adjusted prediction equations and robust z-score-based reference ranges for diverse pediatric populations.

## Data Availability

The raw data supporting the conclusions of this article will be made available by the authors, without undue reservation.
