# Supplementary material for: Impulse oscillometry values calibrated against spirometric obstruction in children with suspected asthma
Source: Front Pediatr. 2026 Jun 8;14:1746282. doi: 10.3389/fped.2026.1746282 (PMC13284066; doi:10.3389/fped.2026.1746282)
Supplement: Supplementary file 1 [file Supplementaryfile1.docx]

Supplementary Material

# Supplementary Tables

# Supplementary Table 1. Correlations between post-bronchodilator changes in IOS parameters and spirometry indices in children aged 6–11 years.

| Age: 6-11 | (n=569) | FVC | PEF | FEV1 | FEV1/FVC | MMEF | MEF25 | MEF50 | MEF75 | Change%-FEV1 |
| --- | --- | --- | --- | --- | --- | --- | --- | --- | --- | --- |
| change%-R5 | *ρ* | -0.059 | 0.047 | 0.079 | 0.207† | 0.221† | 0.210† | 0.221† | 0.132** | -0.215† |
|  | (95% CI) | (-0.140, 0.024) | (-0.035, 0.129) | (-0.003, 0.160) | (0.127, 0.284) | (0.141, 0.298) | (0.130, 0.287) | (0.141, 0.298) | (0.050, 0.212) | (-0.311, -0.115) |
| change%-R10 | *ρ* | -0.061 | 0.013 | 0.054 | 0.168† | 0.189† | 0.175† | 0.192† | 0.098* | -0.193† |
|  | (95% CI) | (-0.142, 0.022) | (-0.069, 0.095) | (-0.028, 0.136) | (0.087, 0.247) | (0.108, 0.267) | (0.094, 0.254) | (0.112, 0.270) | (0.016, 0.179) | (-0.290, -0.092) |
| change%-R5 − R20 | *ρ* | -0.054 | 0.093* | 0.073 | 0.204† | 0.195† | 0.190† | 0.195† | 0.137** | -0.143** |
|  | (95% CI) | (-0.136, 0.028) | (0.011, 0.174) | (-0.009, 0.154) | (0.124, 0.281) | (0.115, 0.273) | (0.11, 0.268) | (0.115, 0.273) | (0.055, 0.217) | (-0.242, -0.041) |
| change%-X5 | *ρ* | -0.045 | 0.075 | 0.071 | 0.182† | 0.180† | 0.185† | 0.165† | 0.129** | -0.168** |
|  | (95% CI) | (-0.126, 0.038) | (-0.007, 0.156) | (-0.012, 0.152) | (0.101, 0.260) | (0.099, 0.258) | (0.104, 0.263) | (0.084, 0.244) | (0.047, 0.209) | (-0.266, -0.066) |
| change%-Fres | *ρ* | -0.067 | 0.046 | 0.075 | 0.211† | 0.204† | 0.214† | 0.195† | 0.103* | -0.112* |
|  | (95% CI) | (-0.148, 0.015) | (-0.037, 0.127) | (-0.007, 0.157) | (0.131, 0.288) | (0.124, 0.281) | (0.134, 0.291) | (0.115, 0.273) | (0.021, 0.184) | (-0.212, -0.009) |
| change%-AX | *ρ* | -0.087* | 0.110** | 0.093* | 0.283† | 0.271† | 0.274† | 0.262† | 0.187† | -0.195† |
|  | (95% CI) | (-0.168, -0.005) | (0.028, 0.19) | (0.011, 0.174) | (0.206, 0.357) | (0.193, 0.346) | (0.196, 0.348) | (0.184, 0.337) | (0.106, 0.265) | (-0.292, -0.094) |

# Notes: *ρ* = Spearman's rank correlation coefficient; ^*^, ^**^, and ^†^ represent significance levels of *p* <0.05, *p* <0.01, and *p* <0.001, respectively.

# Supplementary Table 2. Correlations between post-bronchodilator changes in IOS parameters and spirometry indices in children aged 12–18 years.

| Age: 12-18 | (n=153) | FVC | PEF | FEV1 | FEV1/FVC | MMEF | MEF25 | MEF50 | MEF75 | Change%-FEV1 |
| --- | --- | --- | --- | --- | --- | --- | --- | --- | --- | --- |
| change%-R5 | *ρ* | 0.061 | 0.236** | 0.191* | 0.199* | 0.32† | 0.278† | 0.299† | 0.290† | -0.270* |
|  | (95% CI) | (-0.099, 0.218) | (0.080, 0.380) | (0.033, 0.339) | (0.042, 0.347) | (0.170, 0.456) | (0.125, 0.418) | (0.147, 0.437) | (0.138, 0.429) | (-0.454, -0.064) |
| change%-R10 | *ρ* | 0.063 | 0.175* | 0.151 | 0.125 | 0.259** | 0.226** | 0.237** | 0.221** | -0.218* |
|  | (95% CI) | (-0.097, 0.219) | (0.017, 0.325) | (-0.008, 0.302) | (-0.034, 0.279) | (0.105, 0.401) | (0.070, 0.371) | (0.081, 0.381) | (0.065, 0.367) | (-0.409, -0.009) |
| change%-R5− R20 | *ρ* | -0.024 | 0.177* | 0.063 | 0.045 | 0.146 | 0.088 | 0.162* | 0.206* | -0.141 |
|  | (95% CI) | (-0.182, 0.135) | (0.019, 0.327) | (-0.096, 0.220) | (-0.115, 0.202) | (-0.013, 0.298) | (-0.072, 0.243) | (0.003, 0.313) | (0.049, 0.353) | (-0.341, 0.070) |
| change%-X5 | *ρ* | 0.053 | 0.233** | 0.234** | 0.252** | 0.337† | 0.319† | 0.302† | 0.31† | -0.211* |
|  | (95% CI) | (-0.106, 0.210) | (0.077, 0.378) | (0.078, 0.379) | (0.097, 0.395) | (0.188, 0.471) | (0.169, 0.455) | (0.151, 0.440) | (0.159, 0.447) | (-0.403, -0.002) |
| change%-Fres | *ρ* | 0.062 | 0.250** | 0.255** | 0.308† | 0.345† | 0.334† | 0.335† | 0.352† | -0.313** |
|  | (95% CI) | (-0.098, 0.218) | (0.095, 0.393) | (0.100, 0.398) | (0.157, 0.445) | (0.197, 0.478) | (0.185, 0.468) | (0.186, 0.469) | (0.205, 0.484) | (-0.490, -0.111) |
| change%-AX | *ρ* | 0.058 | 0.275† | 0.274† | 0.318† | 0.399† | 0.380† | 0.369† | 0.370† | -0.304** |
|  | (95% CI) | (-0.102, 0.214) | (0.122, 0.416) | (0.121, 0.415) | (0.168, 0.454) | (0.257, 0.524) | (0.236, 0.508) | (0.223, 0.498) | (0.224, 0.499) | (-0.483, -0.101) |

# Notes: *ρ* = Spearman's rank correlation coefficient; ^*^, ^**^, and ^†^ represent significance levels of *p* <0.05, *p* <0.01, and *p* <0.001, respectively.

**Supplementary Table 3.** Accuracy and likelihood ratios for various IOS cut-off values relative to spirometry-defined airway obstruction in children aged 6–11 and 12–18 years.

| Age | Variable | Cut-off value | LR (+) | LR (-) | Accuracy (95% CI) |
| --- | --- | --- | --- | --- | --- |
| 6–11 years | R5 (% predicted) | > 150% | 4.185 | 0.992 | 0.678 (0.639-0.715) |
|  | X5 (% predicted) | > 150% | 8.370 | 0.981 | 0.682 (0.643-0.719) |
|  | R5 decrease* | > 20% | 1.551 | 0.844 | 0.638 (0.598-0.676) |
|  | X5 decrease* | > 20% | 1.407 | 0.825 | 0.610 (0.569-0.649) |
|  | R5 − R20 decrease* | > 40% | 1.349 | 0.866 | 0.610 (0.569-0.649) |
|  | Fres decrease* | > 20% | 1.469 | 0.827 | 0.622 (0.582-0.661) |
|  | AX decrease* | > 40% | 1.452 | 0.747 | 0.602 (0.561-0.642) |
|  | AX (kPa·L⁻¹) | ≥1.535 | 2.043 | 0.493 | 0.671 (0.632–0.709) |
|  | Fres (Hz) | ≥20.405 | 2.004 | 0.551 | 0.668 (0.628–0.705) |
|  | R5–R20 (kPa·L⁻¹·s) | ≥0.185 | 1.951 | 0.498 | 0.673 (0.634–0.710) |
| 12–18 years | R5 (% predicted) | > 150% | 4.875 | 0.933 | 0.771 (0.699-0.831) |
|  | X5 (% predicted) | > 150% | 4.875 | 0.933 | 0.771 (0.699-0.831) |
|  | R5 decrease* | > 20% | 3.250 | 0.669 | 0.765 (0.692-0.825) |
|  | X5 decrease* | > 20% | 1.920 | 0.787 | 0.706 (0.629-0.772) |
|  | R5 − R20 decrease* | > 40% | 1.767 | 0.535 | 0.633 (0.554-0.706) |
|  | Fres decrease* | > 20% | 2.511 | 0.650 | 0.732 (0.657-0.796) |
|  | AX decrease* | > 40% | 3.039 | 0.528 | 0.757 (0.683-0.818) |
|  | AX (kPa·L⁻¹) | ≥0.538 | 2.000 | 0.500 | 0.667 (0.589–0.736) |
|  | Fres (Hz) | ≥13.772 | 1.755 | 0.437 | 0.614 (0.535–0.688) |
|  | R5–R20 (kPa·L⁻¹·s) | ≥0.062 | 1.867 | 0.418 | 0.634 (0.555–0.706) |

# Note: *indicates a decrease in absolute difference before and after inhalation of bronchodilators, regardless of sign.

**Supplementary Table 4.** Supplementary data on diagnosing obstructive spirometry accuracy from IOS changes before and after bronchodilator inhalation for children aged 6 to 11 years.

|  | | Normal  N (%) | Abnormal  N (%) | Total  N (%) | *p*-value  (FDR) | Sensitivity  (95% CI) | Specificity  (95% CI) | PPV  (95% CI) | NPV  (95% CI) | Accuracy  (95% CI) |
| --- | --- | --- | --- | --- | --- | --- | --- | --- | --- | --- |
| R5 decrease >40% | No | 383 (99.5) | 183 (99.5) | 566 (99.5) | >0.999 | 0.663 (0.592-0.727) | 0.497 (0.448-0.547) | 0.387 (0.335-0.442) | 0.755 (0.698-0.804) | 0.551 (0.510-0.591) |
|  | Yes | 2 (0.5) | 1 (0.5) | 3 (0.5) |  | 0.810 (0.747-0.860) | 0.338 (0.292-0.386) | 0.369 (0.323-0.417) | 0.788 (0.719-0.843) | 0.490 (0.449-0.531) |
| R5 decrease >10% | No | 164 (42.6) | 51 (27.7) | 215 (37.8) | 0.001 | 0.707 (0.637-0.768) | 0.460 (0.411-0.510) | 0.385 (0.334-0.438) | 0.766 (0.708-0.816) | 0.540 (0.498-0.580) |
|  | Yes | 221 (57.4) | 133 (72.3) | 35 (62.2) |  | 0.723 (0.654-0.782) | 0.426 (0.378-0.476) | 0.376 (0.327-0.427) | 0.763 (0.702-0.815) | 0.522 (0.481-0.563) |
| X5 decrease >40% | No | 362 (94.0) | 166 (90.2) | 528 (92.8) | 0.117 | 0.005 (0.001-0.030) | 0.995 (0.981-0.999) | 0.333 (0.061-0.792) | 0.677 (0.637-0.714) | 0.675 (0.635-0.712) |
|  | Yes | 23 (6.0) | 18 (9.8) | 41 (7.2) |  | 0.652 (0.581-0.717) | 0.496 (0.446-0.546) | 0.382 (0.330-0.437) | 0.749 (0.692-0.798) | 0.547 (0.505-0.587) |
| X5 decrease >10% | No | 191 (49.6) | 64 (34.8) | 255 (44.8) | 0.001 | 0.098 (0.063-0.149) | 0.940 (0.912-0.960) | 0.439 (0.299-0.590) | 0.686 (0.645-0.724) | 0.668 (0.628-0.705) |
|  | Yes | 194 (50.4) | 120 (65.2) | 314 (55.2) |  | 0.663 (0.592-0.727) | 0.497 (0.448-0.547) | 0.387 (0.335-0.442) | 0.755 (0.698-0.804) | 0.551 (0.510-0.591) |
| R5 − R20 decrease >20% | No | 177 (46.0) | 54 (29.3) | 231 (40.6) | 0.001 | 0.810 (0.747-0.860) | 0.338 (0.292-0.386) | 0.369 (0.323-0.417) | 0.788 (0.719-0.843) | 0.490 (0.449-0.531) |
|  | Yes | 208 (54.0) | 130 (70.7) | 338 (59.4) |  | 0.707 (0.637-0.768) | 0.460 (0.411-0.510) | 0.385 (0.334-0.438) | 0.766 (0.708-0.816) | 0.540 (0.498-0.580) |
| R5 − R20 decrease >10% | No | 130 (33.8) | 35 (19.0) | 165 (29.0) | 0.001 | 0.723 (0.654-0.782) | 0.426 (0.378-0.476) | 0.376 (0.327-0.427) | 0.763 (0.702-0.815) | 0.522 (0.481-0.563) |
|  | Yes | 255 (66.2) | 149 (81.0) | 404 (71.0) |  | 0.005 (0.001-0.030) | 0.995 (0.981-0.999) | 0.333 (0.061-0.792) | 0.677 (0.637-0.714) | 0.675 (0.635-0.712) |
| AX decrease >30% | No | 191 (49.7) | 62 (33.7) | 253 (44.5) | 0.001 | 0.652 (0.581-0.717) | 0.496 (0.446-0.546) | 0.382 (0.330-0.437) | 0.749 (0.692-0.798) | 0.547 (0.505-0.587) |
|  | Yes | 193 (50.3) | 122 (66.3) | 315 (55.5) |  | 0.098 (0.063-0.149) | 0.940 (0.912-0.960) | 0.439 (0.299-0.590) | 0.686 (0.645-0.724) | 0.668 (0.628-0.705) |

# Note: *p*-values were calculated using the Chi-square test or Fisher’s exact test, depending on cell counts, and were adjusted for multiple comparisons using the Benjamini-Hochberg false discovery rate (FDR) correction.

**Supplementary Table 5.** Supplementary data on diagnosing obstructive spirometry accuracy from IOS changes before and after bronchodilator inhalation for children aged 12 to 18 years.

|  | | Normal  N (%) | Abnormal  N (%) | Total  N (%) | *p*-value  (FDR) | Sensitivity  (95% CI) | Specificity  (95% CI) | PPV  (95% CI) | NPV  (95% CI) | Accuracy  (95% CI) |
| --- | --- | --- | --- | --- | --- | --- | --- | --- | --- | --- |
| R5 decrease >40% | No | 117 (100.0) | 35 (97.2) | 152 (99.3) | 0.275 | 0.028 | 1.000 | 1.000 | 0.770 | 0.771 |
|  | Yes | 0 (0.0) | 1 (2.8) | 1 (0.7) |  | (0.005-0.142) | (0.968-1.000) | (0.207-1.000) | (0.697-0.830) | (0.699-0.831) |
| R5 decrease >10% | No | 58 (49.6) | 9 (25.0) | 67 (43.8) | 0.017 | 0.750 | 0.496 | 0.314 | 0.866 | 0.556 |
|  | Yes | 59 (50.4) | 27 (75.0) | 86 (56.2) |  | (0.589-0.862) | (0.407-0.585) | (0.226-0.418) | (0.764-0.928) | (0.476-0.632) |
| X5 decrease >40% | No | 113 (96.6) | 34 (94.4) | 147 (96.1) | 0.626 | 0.056 | 0.966 | 0.333 | 0.769 | 0.752 |
|  | Yes | 4 (3.4) | 2 (5.6) | 6 (3.9) |  | (0.015-0.181) | (0.915-0.987) | (0.097-0.700) | (0.694-0.830) | (0.678-0.813) |
| X5 decrease >10% | No | 77 (65.8) | 15 (41.7) | 92 (60.1) | 0.017 | 0.583 | 0.658 | 0.344 | 0.837 | 0.641 |
|  | Yes | 40 (34.2) | 21 (58.3) | 61 (39.9) |  | (0.422-0.729) | (0.568-0.738) | (0.237-0.470) | (0.748-0.899) | (0.562-0.712) |
| R5 − R20 decrease >20% | No | 50 (43.9) | 7 (19.4) | 57 (38.0) | 0.017 | 0.806 | 0.439 | 0.312 | 0.877 | 0.527 |
|  | Yes | 64 (56.1) | 29 (80.6) | 93 (62.0) |  | (0.650-0.902) | (0.351-0.530) | (0.227-0.412) | (0.768-0.939) | (0.447-0.605) |
| R5 − R20 decrease >10% | No | 41 (36.0) | 5 (13.9) | 46 (30.7) | 0.017 | 0.861 | 0.360 | 0.298 | 0.891 | 0.480 |
|  | Yes | 73 (64.0) | 31 (86.1) | 104 (69.3) |  | (0.713-0.939) | (0.277-0.451) | (0.219-0.392) | (0.770-0.953) | (0.402-0.559) |
| AX decrease >30% | No | 80 (68.4) | 10 (28.6) | 90 (59.2) | <0.001 | 0.714 | 0.684 | 0.403 | 0.889 | 0.691 |
|  | Yes | 37 (31.6) | 25 (71.4) | 62 (40.8) |  | (0.549-0.837) | (0.595-0.761) | (0.290-0.527) | (0.807-0.939) | (0.613-0.759) |

# Note: *p* -values were calculated using the Chi-square test or Fisher’s exact test, depending on cell counts, and were adjusted for multiple comparisons using the Benjamini-Hochberg false discovery rate (FDR) correction.

**Supplementary Table 6.** Distribution of spirometry-defined functional subgroups and bronchodilator responsiveness subgroups across two age groups.

|  |  | Total | | Age | | | | *p*-value |
| --- | --- | --- | --- | --- | --- | --- | --- | --- |
|  |  |  |  | 6-11 | | 12-18 | |  |
|  |  | N | % | N | % | N | % |  |
| LAO-only | No | 623 | 86.3 | 494 | 86.8 | 129 | 84.3 | 0.424 |
|  | Yes | 99 | 13.7 | 75 | 13.2 | 24 | 15.7 |  |
| SAO-only | No | 621 | 86.0 | 481 | 84.5 | 140 | 91.5 | 0.027 |
|  | Yes | 101 | 14.0 | 88 | 15.5 | 13 | 8.5 |  |
| Combined obstruction | No | 628 | 87.0 | 490 | 86.1 | 138 | 90.2 | 0.183 |
|  | Yes | 94 | 13.0 | 79 | 13.9 | 15 | 9.8 |  |
| BDR-FEV₁ positive | No | 695 | 96.3 | 547 | 96.1 | 148 | 96.7 | 0.729 |
|  | Yes | 27 | 3.7 | 22 | 3.9 | 5 | 3.3 |  |
| BDR-FEF positive | No | 600 | 83.1 | 468 | 82.2 | 132 | 86.3 | 0.238 |
|  | Yes | 122 | 16.9 | 101 | 17.8 | 21 | 13.7 |  |
| Overall BDR positive | No | 596 | 82.5 | 465 | 81.7 | 131 | 85.6 | 0.259 |
|  | Yes | 126 | 17.5 | 104 | 18.3 | 22 | 14.4 |  |

# Notes: (1) Large airway obstruction only (LAO-only): PEFR, FEV₁, or FEV₁/FVC < 80% of predicted, while all FEF parameters are ≥ 56% of predicted; (2) Small airway obstruction only (SAO-only): PEFR, FEV₁, and FEV₁/FVC are all ≥ 80% of predicted, but at least one of the FEF parameters is < 56% of predicted; (3) Combined obstruction: At least one of PEFR, FEV₁, or FEV₁/FVC is < 80% of predicted, and at least one FEF parameter is < 56% of predicted; (4) BDR-FEV₁ positive: A positive bronchodilator response defined as an increase of ≥ 12% in FEV₁; (5) BDR-FEF positive: A positive bronchodilator response defined as an increase of ≥ 40% in any FEF parameter; (6) Overall BDR positive: A positive bronchodilator response defined as an increase of ≥12% in FEV₁ or ≥ 40% in any FEF parameter; *p*-values were calculated using the Chi-square test.

**Supplementary Table 7.** Bronchodilator responsiveness across spirometry-defined functional subgroups.

| Age |  |  | Total | | LAO-only | | | | *p*-value | SAO-only | | | | *p*-value | Combined obstruction | | | | *p*-value |
| --- | --- | --- | --- | --- | --- | --- | --- | --- | --- | --- | --- | --- | --- | --- | --- | --- | --- | --- | --- |
|  |  |  |  |  | No | | Yes | |  | No | | Yes | |  | No | | Yes | |  |
|  |  |  | N | % | N | % | N | % |  | N | % | N | % |  | N | % | N | % |  |
| 6-11 | BDR-FEV₁ positive | No | 547 | 96.1 | 476 | 96.4 | 71 | 94.7 | 0.515 | 461 | 95.8 | 86 | 97.7 | 0.555 | 483 | 98.6 | 64 | 81.0 | <0.001 |
|  |  | Yes | 22 | 3.9 | 18 | 3.6 | 4 | 5.3 |  | 20 | 4.2 | 2 | 2.3 |  | 7 | 1.4 | 15 | 19.0 |  |
|  | Total |  | 569 | 100.0 | 494 | 100.0 | 75 | 100.0 |  | 481 | 100.0 | 88 | 100.0 |  | 490 | 100.0 | 79 | 100.0 |  |
| 12-18 | BDR-FEV₁ positive | No | 148 | 96.7 | 124 | 96.1 | 24 | 100.0 | >0.999 | 137 | 97.9 | 11 | 84.6 | 0.058 | 136 | 98.6 | 12 | 80.0 | 0.007 |
|  |  | Yes | 5 | 3.3 | 5 | 3.9 | 0 | 0.0 |  | 3 | 2.1 | 2 | 15.4 |  | 2 | 1.4 | 3 | 20.0 |  |
|  | Total |  | 153 | 100.0 | 129 | 100.0 | 24 | 100.0 |  | 140 | 100.0 | 13 | 100.0 |  | 138 | 100.0 | 15 | 100.0 |  |
| Total | BDR-FEV₁ positive | No | 695 | 96.3 | 600 | 96.3 | 95 | 96.0 | 0.778 | 598 | 96.3 | 97 | 96.0 | 0.782 | 619 | 98.6 | 76 | 80.9 | <0.001 |
|  |  | Yes | 27 | 3.7 | 23 | 3.7 | 4 | 4.0 |  | 23 | 3.7 | 4 | 4.0 |  | 9 | 1.4 | 18 | 19.1 |  |
|  | Total |  | 722 | 100.0 | 623 | 100.0 | 99 | 100.0 |  | 621 | 100.0 | 101 | 100.0 |  | 628 | 100.0 | 94 | 100.0 |  |
| 6-11 | BDR-FEF positive | No | 468 | 82.2 | 402 | 81.4 | 66 | 88.0 | 0.162 | 406 | 84.4 | 62 | 70.5 | 0.002 | 423 | 86.3 | 45 | 57.0 | <0.001 |
|  |  | Yes | 101 | 17.8 | 92 | 18.6 | 9 | 12.0 |  | 75 | 15.6 | 26 | 29.5 |  | 67 | 13.7 | 34 | 43.0 |  |
|  | Total |  | 569 | 100.0 | 494 | 100.0 | 75 | 100.0 |  | 481 | 100.0 | 88 | 100.0 |  | 490 | 100.0 | 79 | 100.0 |  |
| 12-18 | BDR-FEF positive | No | 132 | 86.3 | 110 | 85.3 | 22 | 91.7 | 0.532 | 123 | 87.9 | 9 | 69.2 | 0.082 | 125 | 90.6 | 7 | 46.7 | <0.001 |
|  |  | Yes | 21 | 13.7 | 19 | 14.7 | 2 | 8.3 |  | 17 | 12.1 | 4 | 30.8 |  | 13 | 9.4 | 8 | 53.3 |  |
|  | Total |  | 153 | 100.0 | 129 | 100.0 | 24 | 100.0 |  | 140 | 100.0 | 13 | 100.0 |  | 138 | 100.0 | 15 | 100.0 |  |
| Total | BDR-FEF positive | No | 600 | 83.1 | 512 | 82.2 | 88 | 88.9 | 0.098 | 529 | 85.2 | 71 | 70.3 | <0.001 | 548 | 87.3 | 52 | 55.3 | <0.001 |
|  |  | Yes | 122 | 16.9 | 111 | 17.8 | 11 | 11.1 |  | 92 | 14.8 | 30 | 29.7 |  | 80 | 12.7 | 42 | 44.7 |  |
|  | Total |  | 722 | 100.0 | 623 | 100.0 | 99 | 100.0 |  | 621 | 100.0 | 101 | 100.0 |  | 628 | 100.0 | 94 | 100.0 |  |
| 6-11 | Overall BDR positive | No | 465 | 81.7 | 401 | 81.2 | 64 | 85.3 | 0.385 | 403 | 83.8 | 62 | 70.5 | 0.003 | 421 | 85.9 | 44 | 55.7 | <0.001 |
|  |  | Yes | 104 | 18.3 | 93 | 18.8 | 11 | 14.7 |  | 78 | 16.2 | 26 | 29.5 |  | 69 | 14.1 | 35 | 44.3 |  |
|  | Total |  | 569 | 100.0 | 494 | 100.0 | 75 | 100.0 |  | 481 | 100.0 | 88 | 100.0 |  | 490 | 100.0 | 79 | 100.0 |  |
| 12-18 | Overall BDR positive | No | 131 | 85.6 | 109 | 84.5 | 22 | 91.7 | 0.530 | 123 | 87.9 | 8 | 61.5 | 0.023 | 124 | 89.9 | 7 | 46.7 | <0.001 |
|  |  | Yes | 22 | 14.4 | 20 | 15.5 | 2 | 8.3 |  | 17 | 12.1 | 5 | 38.5 |  | 14 | 10.1 | 8 | 53.3 |  |
|  | Total |  | 153 | 100.0 | 129 | 100.0 | 24 | 100.0 |  | 140 | 100.0 | 13 | 100.0 |  | 138 | 100.0 | 15 | 100.0 |  |
| Total | Overall BDR positive | No | 596 | 82.5 | 510 | 81.9 | 86 | 86.9 | 0.223 | 526 | 84.7 | 70 | 69.3 | <0.001 | 545 | 86.8 | 51 | 54.3 | <0.001 |
|  |  | Yes | 126 | 17.5 | 113 | 18.1 | 13 | 13.1 |  | 95 | 15.3 | 31 | 30.7 |  | 83 | 13.2 | 43 | 45.7 |  |
|  | Total |  | 722 | 100.0 | 623 | 100.0 | 99 | 100.0 |  | 621 | 100.0 | 101 | 100.0 |  | 628 | 100.0 | 94 | 100.0 |  |

# Notes: Definitions of spirometry-defined functional subgroups and bronchodilator responsiveness subgroups are provided in the footnotes of Supplementary Table 8; *p*-values were calculated using the chi-square or Fisher’s exact test, as appropriate.

**Supplementary Table 8.** A multivariable logistic regression model combining AX, Fres, and R5-R20 was evaluated in both age strata.

| Age | Model type | Predictors | AUC  (95% CI) | Sensitivity  (95% CI) | Specificity  (95% CI) | PPV  (95% CI) | NPV  (95% CI) | Accuracy  (95% CI) |
| --- | --- | --- | --- | --- | --- | --- | --- | --- |
| 6–11 years | Best single | AX (1.535) | 0.721  (0.678–0.765) | 0.668  (0.598–0.732) | 0.673  (0.624–0.718) | 0.494  (0.432–0.556) | 0.809  (0.763–0.849) | 0.671  (0.632–0.709) |
|  | Multivariable | AX + Fres + R5-R20 | 0.721  (0.677–0.764) | 0.717  (0.648–0.777) | 0.626  (0.577–0.673) | 0.478  (0.420–0.537) | 0.823  (0.775–0.862) | 0.656  (0.616–0.693) |
| 12–18 years | Best single | R5-R20 (0.062) | 0.707  (0.607–0.806) | 0.750  (0.589–0.862) | 0.598  (0.508–0.683) | 0.365  (0.264–0.479) | 0.886  (0.797–0.939) | 0.634  (0.555–0.706) |
|  | Multivariable | AX + Fres + R5-R20 | 0.709  (0.608–0.810) | 0.556  (0.396–0.705) | 0.795  (0.713–0.858) | 0.455  (0.317–0.599) | 0.853  (0.775–0.908) | 0.739  (0.664–0.802) |

## Supplementary Table 9. Adjusted multivariable logistic regression results for children aged 6-11 years.

| Variable | Cutoff (Youden) | AUC  (Crude) | 95% CI  (Crude) | AUC  (Adjusted) | 95% CI  (Adjusted) | OR  (Adjusted) | 95% CI  (Adjusted OR) | *p*-value |
| --- | --- | --- | --- | --- | --- | --- | --- | --- |
| R5–R20 (kPa·L⁻¹·s) | 0.185 | 0.714 | 0.670–0.758 | 0.707 | 0.662–0.752 | 5.03 | 3.33–7.71 | <0.001 |
| AX (kPa·L⁻¹) | 1.535 | 0.721 | 0.678–0.765 | 0.711 | 0.665–0.756 | 5.28 | 3.47–8.17 | <0.001 |
| Fres (Hz) | 20.405 | 0.704 | 0.659–0.749 | 0.692 | 0.646–0.738 | 4.19 | 2.81–6.30 | <0.001 |

# Notes: (1) Model type: Multivariable logistic regression adjusted for age, BMI, height, and sex; (2) AUC: Area under the ROC curve; (3) 95% confidence intervals (CIs) were estimated using the Wilson method; (4) Adjusted OR: Odds ratio from the multivariable logistic regression model, indicating the adjusted odds of having obstructive spirometric pattern per diagnostic cutoff; (5) All *p*-values < 0.001 indicate statistical significance after adjustment.

## Supplementary Table 10. Internal and temporal validation of diagnostic models for children in both age strata were performed using 10-fold cross-validation and 1,000 bootstrap resamples.

| Age | Variable | AUC  (Original) | 95% CI  (Original) | AUC  (Corrected) | 95% CI  (Corrected) | ΔAUC | Comment |
| --- | --- | --- | --- | --- | --- | --- | --- |
| 6–11 years | AX (kPa·L⁻¹) | 0.721 | 0.678–0.765 | — | — | — |  |
|  | Fres (Hz) | 0.704 | 0.659–0.749 | — | — | — |  |
|  | R5–R20 (kPa·L⁻¹·s) | 0.714 | 0.670–0.758 | — | — | — |  |
|  | Multivariable | 0.721 | 0.677–0.764 | 0.723 | 0.680–0.765 | +0.002 | Excellent stability after bootstrap |
| 12–18 years | AX (kPa·L⁻¹) | 0.677 | 0.568–0.787 | — | — | — |  |
|  | Fres (Hz) | 0.678 | 0.573–0.782 | — | — | — |  |
|  | R5–R20 (kPa·L⁻¹·s) | 0.707 | 0.607–0.806 | — | — | — |  |
|  | Multivariable | 0.709 | 0.608–0.810 | 0.707 | 0.588–0.806 | −0.002 | Stable, minimal optimism bias |

# Notes: (1) Internal validation was performed using 1,000 bootstrap resamples for optimism correction; (2) Corrected AUCs represent the bootstrap-adjusted (optimism-corrected) performance estimates; (3) ΔAUC = AUC(Corrected) – AUC (Original). Temporal validation showed consistent results (ΔAUC < 0.01); (4) Models remained stable across both age groups, indicating good internal robustness and generalizability.

# Supplementary Table 11. Descriptive statistics of AX, Fres, and R5-R20 in the SAO-only group compared with the Non-obstructive group

|  | Age 6-11 (N=569) | | | | | | Age 12-18 (N=153) | | | | |
| --- | --- | --- | --- | --- | --- | --- | --- | --- | --- | --- | --- |
|  | SAO-only (N=88) | | | No obstruction (N=385) | | *p*-value | SAO-only (N=13) | | No obstruction (N=117) | | *p*-value |
|  | Mean ± SD | | Median (IQR) | Mean ± SD | Median (IQR) |  | Mean ± SD | Median (IQR) | Mean ± SD | Median (IQR) |  |
| AX | | 1.92 ± 1.11 | 1.62 (1.15-2.46) | 1.34 ± 0.89 | 1.15 (0.67-1.78) | <0.001 | 1.18 ± 0.73 | 1.22 (0.68-1.47) | 0.56 ± 0.4 | 0.44 (0.27-0.64) | 0.001 |
| Fres | | 20.82 ± 3.72 | 20.47 (18.24-23.59) | 18.3 ± 4.32 | 18.53 (15.43-21.26) | <0.001 | 18.38 ± 4.28 | 18.65 (15.24-21.76) | 13.81 ± 3.9 | 13.02 (10.58-16.37) | 0.001 |
| R5-R20Hz | | 0.21 ± 0.09 | 0.21 (0.14-0.26) | 0.15 ± 0.09 | 0.14 (0.09-0.21) | <0.001 | 0.15 ± 0.08 | 0.16 (0.07-0.20) | 0.06 ± 0.06 | 0.05 (0.02-0.1) | <0.001 |

# Abbreviations: SAO= small airway obstruction; SD = standard deviation; IQR = interquartile range represents the distance between the 25th percentile and 75th percentile.

Note: *p*-values were calculated using the Mann-Whitney U Test.
